# Emerging Green Techniques for the Extraction of Antioxidants from Agri-Food By-Products as Promising Ingredients for the Food Industry

**DOI:** 10.3390/antiox10091417

**Published:** 2021-09-05

**Authors:** Serena Carpentieri, Farid Soltanipour, Giovanna Ferrari, Gianpiero Pataro, Francesco Donsì

**Affiliations:** 1 Department of Industrial Engineering, University of Salerno, via Giovanni Paolo II, 132, 84084 Fisciano, Italy; scarpentieri@unisa.it (S.C.); fsoltanipour@unisa.it (F.S.); gferrari@unisa.it (G.F.); gpataro@unisa.it (G.P.); 2 ProdAl Scarl, University of Salerno, via Giovanni Paolo II, 132, 84084 Fisciano, Italy

**Keywords:** green extraction, emerging technologies, solvent selection criteria, process sustainability, mass transfer resistances, biorefinery

## Abstract

Nowadays, the food industry is heavily involved in searching for green sources of valuable compounds, to be employed as potential food ingredients, to cater to the evolving consumers’ requirements for health-beneficial food ingredients. In this frame, agri-food by-products represent a low-cost source of natural bioactive compounds, including antioxidants. However, to effectively recover these intracellular compounds, it is necessary to reduce the mass transfer resistances represented by the cellular envelope, within which they are localized, to enhance their extractability. To this purpose, emerging extraction technologies, have been proposed, including Supercritical Fluid Extraction, Microwave-Assisted Extraction, Ultrasound-Assisted Extraction, High-Pressure Homogenization, Pulsed Electric Fields, High Voltage Electrical Discharges. These technologies demonstrated to be a sustainable alternative to conventional extraction, showing the potential to increase the extraction yield, decrease the extraction time and solvent consumption. Additionally, in green extraction processes, also the contribution of solvent selection, as well as environmental and economic aspects, represent a key factor. Therefore, this review focused on critically analyzing the main findings on the synergistic effect of low environmental impact technologies and green solvents towards the green extraction of antioxidants from food by-products, by discussing the main associated advantages and drawbacks, and the criteria of selection for process sustainability.

## 1. Introduction

The United Nations defined a new sustainability-focused development plan consisting of 17 sustainable goals, recognizing, among others, the needs for sustainable chemistry and engineering [1,2]. The main objective of green chemistry is to reduce the usage of hazardous substances while reducing energy consumption and promoting the use of more renewable sources [1]. In this framework, the solvent selection is a key factor in the sustainable development of processes for the extraction of bioactive compounds from natural sources [3,4]. Solvents define an important part of the performance of industrial extraction processes having an impact on the recovery yield and quality of the extracts, as well as on costs, and environmental issues. Their losses represent a major contribution to pollution of industrial processes, while their purification and recovery claim a large part of their energy consumption [1]. To overcome these issues, greener solvents have been proposed as alternatives to petrochemical solvents, being non-toxic, recyclable, biodegradable, and with a low energy cost of synthesis [5]. They could be grouped into the following categories:

- Neoteric solvents;
- Supercritical fluids;
- Bio-based solvents;
- Supramolecular solvents.

The main advantages and disadvantages associated with each solvent group are summarized in Table 1 and critically discussed throughout the review (Section 3).

Since the food industry is constantly looking for natural ingredients to meet the consumers’ dynamic demands, agri-food by-products and residues, as cost-effective matrices, represent a reasonable source of natural valuable compounds, including antioxidants. Table 2 reports a classification of the main agri-food-products, with the indication of the main antioxidant compounds that can be recovered, and their health-beneficial properties.

The use of green and sustainable solvents, coupled with the use of low environmental impact technologies, represent a promising holistic approach for the development of “green” extraction processes [13]. In this framework, the agri-food industries have strived to improve their process sustainability through strategies based on the implementation of emerging green technologies, to decrease the overall environmental impact and improve the process efficiency for transforming agri-food residues into high value-added products [14].

More specifically, the improvement of process efficiency requires both to maintain a sustained driving force for the extraction process, for example by acting on the affinity and solubilization of the target solutes in the selected solvent, and to reduce the mass transfer resistances, for example through the permeabilization or destruction of the plant cell envelope, which represent the main physical barriers for the diffusion of the target compounds. Thus, the integration of “green” solvents into a biorefinery of agri-food by-products based on innovative technologies can be expected to support the development of truly sustainable extraction processes of natural antioxidant compounds [1,2].

This review aims at providing a comprehensive and up-to-date analysis of the main findings associated with the synergistic effect of emerging technologies and green solvents on the extraction processes of antioxidants from agri-food by-products. It summarizes the current state of knowledge of the topic, by discussing the main advantages and drawbacks presented in recently published papers associated with their application, as well as the criteria of the solvent and technology selection to define an extraction process, which is sustainable and green.

## 2. Emerging Green Technologies and Involved Mechanisms of Cell Disintegration

Mass transfer enhancement represents the challenge for the efficient extraction of antioxidants or other valuable bioactive compounds from biomass. The main mass transfer resistances lay in the membranes that separate the intracellular compounds, such as polyphenols located in vacuoles and chloroplasts of plant cells, from the outside environment, hindering their diffusion [51]. The permeabilization or physical rupture of cell membranes can, therefore, facilitate the diffusion of the target molecules, and thus reduce extraction time and increase process efficiency [52]. Different techniques based on mechanical, chemical, thermal, or electrical methods can be used to affect the cell membrane.

Conventional extraction processes heavily rely on the use of organic solvents, long processing times, high temperatures, and large expenditure of energy that might negatively impact both human health and the environment. To overcome these limitations, innovative processes have been proposed and widely investigated [53].

The most promising innovative extraction techniques include Supercritical Fluid Extraction (SFE), Microwave-Assisted Extraction (MAE), Ultrasound-Assisted Extraction (UAE), High-Pressure Homogenization (HPH), Pulsed Electric Fields (PEF), High Voltage Electrical Discharges (HVED). The main advantages and drawbacks associated with these technologies, according to the different cell rupture mechanisms induced by each technique, are described in the following sections and summarized in Table 3.

### 2.1. Supercritical Fluid Extraction (SFE)

**Figure 1:**
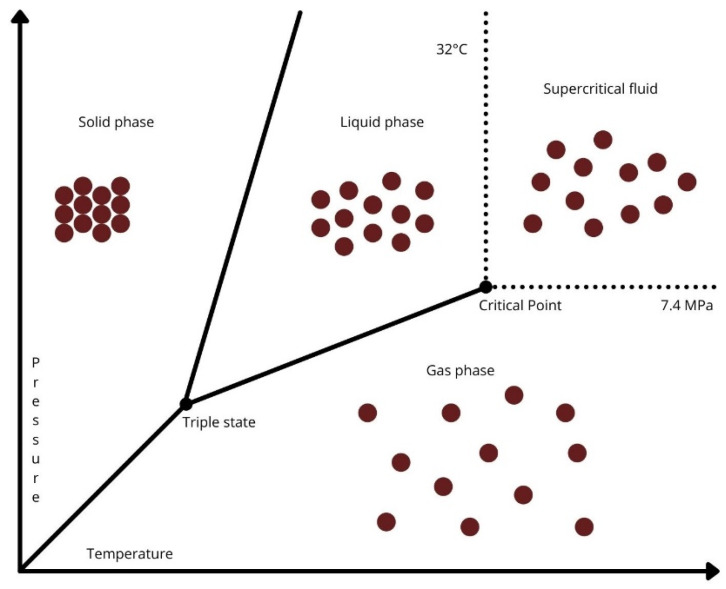
Phase diagram of carbon dioxide.

The physicochemical properties of different solvents vary greatly depending on their phase. The liquid phase has a higher density than the gas phase and therefore has more power to dissolve analytes. On the other hand, the gas phase is more diffusible due to its lower viscosity. The simultaneous presence of these properties, i.e., high liquid-like density and low gas-like viscosity are typically found in a supercritical fluid, which is, therefore, especially suitable for emerging supercritical fluid extraction (SFE) processes, leading to an increase in mass transfer between the target compound and the supercritical fluid [63]. By controlling temperature and pressure, the fluid can be brought in the supercritical region, where the physicochemical properties of the gas and liquid phases can be simultaneously exploited (Figure 1) [64]. Therefore, this aspect offers the possibility of tuning solvent selectivity [63].

The most common solvent used as a supercritical fluid is CO_2_ since it is non-toxic, non-corrosive, non-flammable, inexpensive, and has low viscosity and high diffusivity [64]. In addition, CO_2_ has a low critical temperature and pressure values (31 °C and 74 bar), which is suitable for the extraction of thermo-sensitive molecules [65].

SFE consists of two main stages: extraction and separation. In the extraction part, the fluid is compressed to the required pressure and brought to the required temperature and then diffuses in the sample dissolving the soluble material, transferring it out of the matrix and to the separator section. Temperature and/or pressure can be, then, readjusted to reduce the supercritical fluid solubility, leading to the precipitation of the extracted analytes, in the separation part [64].

In general, SFE is able to increase the extraction yields; at the same time, the absence of organic or toxic solvent residues in the extraction products eliminates further purification steps, which makes it economically acceptable at an industrial scale. However high-pressure equipment and accurate temperature and pressure monitoring need high initial investment on an industrial scale [54,55].

### 2.2. Microwave-Assisted Extraction (MAE)

**Figure 2:**
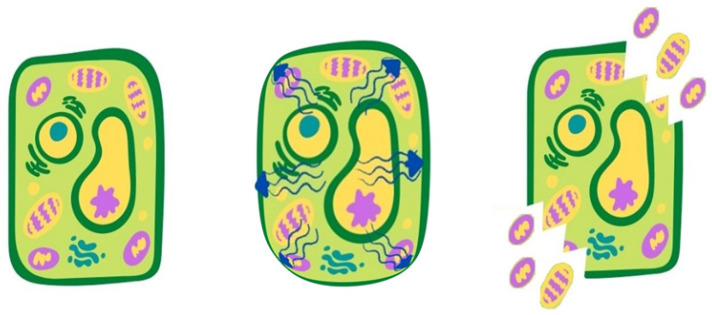
Mechanism of cell rupture induced by microwave: rapid increase of temperature and pressure inside the cell, cell break down and release of target molecules.

Microwave-Assisted Extraction (MAE) is a green extraction technique based on the use of electromagnetic waves that penetrate the product, generate heat inside the matrix, causing the subsequent breakdown of the cell walls [66]. The direct generation of heat during the microwave exposure induces a rise in temperature and pressure of water vapor inside the cell, leading to a swelling of the cell wall up to its rupture and the subsequent release of the intracellular compounds (Figure 2). A faster extraction, with consequent time reduction, can be induced by uniform heating, which will promote the heat and mass transfer in the same direction [23].

Various factors are effective in improving the efficiency of MAE, the most important of which include solvent and matrix properties, temperature, time, and microwave power.

In the solvent selecting step, the characteristics of the microwave adsorption by the solvent, the interaction of the solvent with the matrix, and the solubility of the analyte in the solvent should be considered. The efficiency and selectivity of MAE depend on the solvent dielectric constant. The solvent must have a high dielectric constant and absorb large amounts of microwave energy. Organic solvents such as ethanol, methanol, and water are sufficiently polarized and heated by microwaves.

One of the important parameters in MAE is temperature. In closed system MAE, the temperature may reach above the solvent boiling point. Although higher temperatures improve the extraction efficiency, they can be detrimental to the extraction of thermolabile components.

Microwave power and timing are two mutually dependent factors. Extraction time in MAE usually does not exceed 30 min at 30 W. In general, extraction efficiency is improved when increasing microwave power, and, therefore, extraction time is generally greatly shortened (1 to 2 min at 150 W).

In many cases, extraction efficiency is increased for wet tissues. Matrices containing a high amount of water, which efficiently absorb microwaves energy are subjected to a quick rise in the internal temperature, causing cell disruption, and, therefore, enhancing the extraction of intracellular compounds [67]. Fine particles (100 µm–2 mm) also promote the deeper penetration of the microwave, improving matrix-solvent interaction due to higher surface area [68].

### 2.3. Ultrasound-Assisted Extraction (UAE)

**Figure 3:**
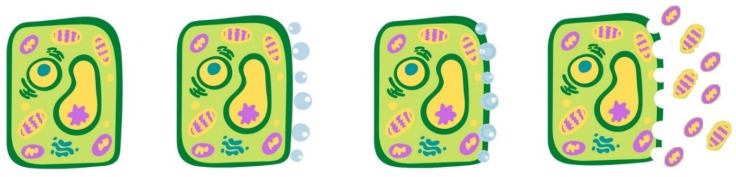
Mechanism of cell rupture induced by ultrasounds: cavitation bubble generation, bubble collapse, and cell wall fragmentation.

In recent years, UAE emerged as an innovative technique, capable of improving heat and mass transfer through the rupture of plant cell walls because of the cavitation effect. The effects of high-power ultrasound on improving extraction are attributed to acoustic cavitation that consists of the formation, growth, and collapse of microbubbles inside a liquid subjected to ultrasonic irradiation (frequencies greater than 20 kHz). This collapse is accompanied by localized high pressures and temperatures, acoustic streaming, high shear stress, microjets near the solid surfaces, and turbulence [31,69]. More specifically, as shown in Figure 3, a cavitation bubble can be generated close to the plant material, then during a compression cycle, this bubble collapse, and a microjet directed toward the plant matrix is created. The high pressure and temperature involved in this process are able to destroy the cell walls, increasing the surface area exposed to the solvent, and, thus, favoring its capillary penetration and promoting the release of the intracellular compounds.

**Figure 4:**
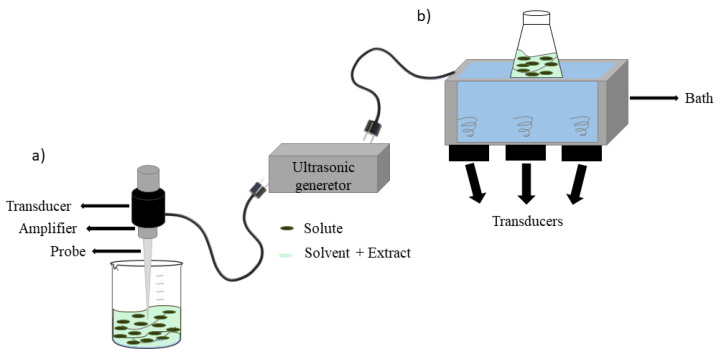
(**a**) Ultrasonic probe system and (**b**) ultrasonic bath system.

The piezoelectric transducer is the basis of both ultrasonic bath and ultrasonic probe systems, which are mainly used in the UAE. Using an ultrasonic bath is easy and affordable, but scale-up and reproducibility are limited. In this system, a stainless-steel tank is connected to a transducer, in which the solid matrix dispersed in the solvent is placed. In contrast, in an ultrasonic probe system, a probe, connected to an ultrasound transducer, is immersed in the extraction vessel, hence minimizing the energy loss in the media (Figure 4). Due to the greater intensity that can be generated in the ultrasonic probe system, this method is usually preferred as a powerful tool in bioactive extraction, because concentrating energy in a specific area of the matrix makes the cavitation effect more efficient [70]. However, the ultrasonic probe system use is limited to a small sample volume. The most important parameter to consider in the UAE at an industrial scale is the product to be treated. It is possible to use a continuous system that can handle a larger amount with a restricted reactor volume or use ultrasonic baths with a larger radiating surface [69].

Effective parameters in UAE are power, temperature, and time of ultrasonic treatment. The effect of these three parameters on the process of extraction yield is similar because, with the increase of each of them, the extraction efficiency also increases. After reaching a maximum point, further increases in each of these parameters will cause the yield reduction. The frequency of ultrasonic is usually comprised between 20 and 120 kHz. Usually, solvents such as acidified water, ethanol, other alcohols, and their solutions are used [71].

### 2.4. Pulsed Electric Fields (PEF)

**Figure 5:**
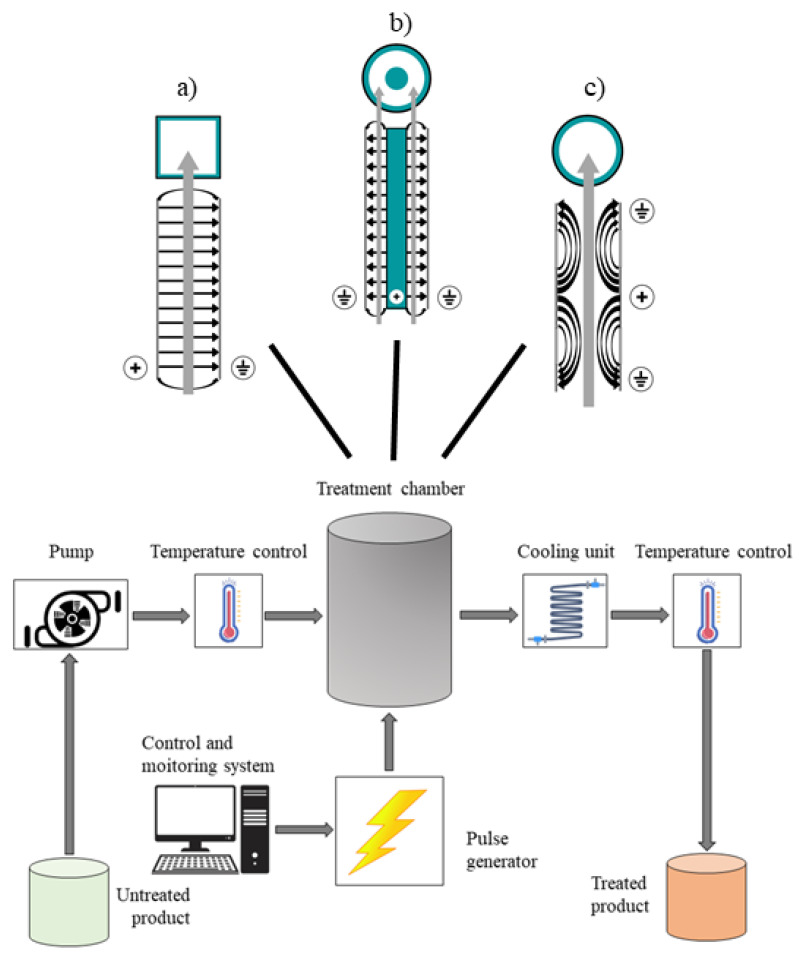
Schematics of a typical continuous flow PEF system, and cross-sectional views of different treatment chamber configurations; (**a**) parallel plate, (**b**) co-axial, (**c**) collinear, (the grey arrows are representative of the product flow).

**Figure 6:**
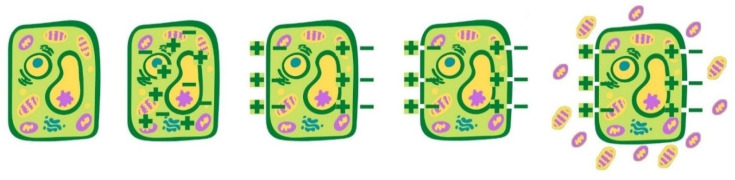
Mechanism of cell disintegration induced by PEF: electroporation phenomenon.

Extraction aided by PEF is an innovative process for the recovery of bioactive compounds from the residues of the agri-food chain [72]. The effectiveness of PEF treatment depends on several parameters, including electric field strength, total specific energy input, and treatment temperature [73]. A typical PEF system comprises a high-voltage pulse generator, a treatment chamber (parallel plate, co-axial, collinear configurations), a pump (for continuous mode operation), a temperature control system, as well as devices like oscilloscope, voltage, and current probes for process monitoring and data acquisition (Figure 5). In PEF processing, a plant matrix in wet form is physically and electrically contacted with the metal electrodes of a treatment chamber, operated either batch-wise or in continuous, and exposed to repetitive (Hz-kHz) very short (μs-ms) electric field (E) pulses of moderate intensity (E = 1–10 kV/cm) and relatively low energy input (W_T_ = 1–20 kJ/kg) supplied by the pulse generator. The pulse shapes commonly used in PEF treatments are either exponential or square-wave pulses, monopolar or bipolar [74]. Depending on the treatment intensity, size, and morphological characteristics of biological cells, the application of electric pulses may cause reversible or irreversible pore formation on the cell membranes, referred to as electroporation or electropermeabilization, as schematized in Figure 6, [74].

This has been proved to improve the efficiency of the conventional extraction processes of valuable compounds from several plant tissues of fruit and vegetable origin, by facilitating the penetration of the solvent into the cells and the selective release of valuable compounds towards the extracting medium [75].

Although the technology is heading for wider industrial application, several limitations still hinder the commercialization of PEF technology. One of the challenges is the development of more reliable and affordable pulse generation systems with sufficient electrical field strength, power, and repetition rate, as well as the optimization of the overall PEF system design, to fulfill current industrial requirements in terms of throughput and treatment uniformity. Moreover, several technological issues, economical pitfalls, consumer acceptance, and regulatory aspects, as well as toxicity risks remain and have to be addressed prior to the full exploitation of PEF technology in different sectors of the food industry [59].

### 2.5. High Voltage Electrical Discharge (HVED)

HVED is a cell disintegration technique of wet biomaterial based on the phenomenon of the electrical breakdown of water. The HVED system includes a high-voltage pulse generator, a treatment chamber an oscilloscope for data acquisition, a cooling system, a peristaltic pump, and voltage and current measuring devices. During an HVED treatment, high energy is directly released into the aqueous suspension placed in a batch treatment chamber between a high voltage needle electrode and a plated grounded electrode through a plasma channel formed by a short duration (2–5 μs) high-current/high-voltage electrical discharge (40–60 kV; 10 kA) [76].

**Figure 7:**
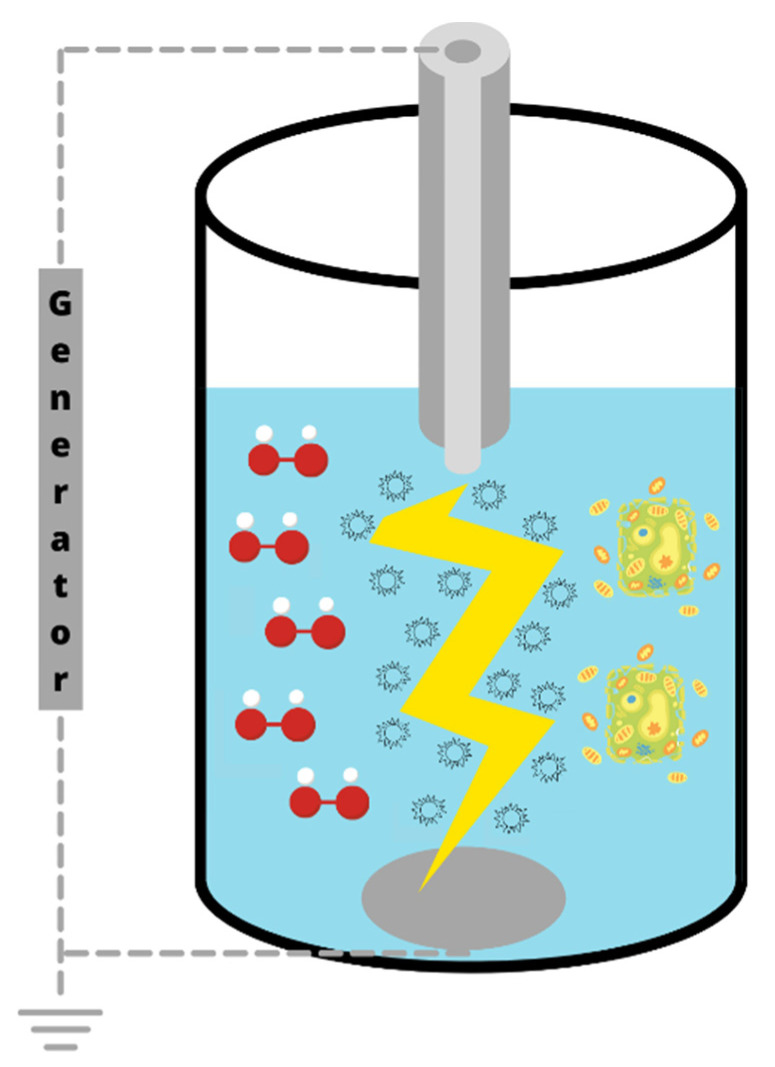
Mechanisms involved in the HVED process, enabling the cell disintegration and the release of target compounds: hydroxyl radical generation, electrical breakdown, collapse of cavitation bubbles, shock waves, and turbulence.

Although the mechanisms of HVED are not yet well understood, the combination of electrical breakdown with different secondary phenomena such as high-amplitude pressure shock waves, bubble cavitation, creation of liquid turbulence, and production of active species, occurring during the treatment, are likely the cause of particle fragmentation and cell structural damages, including cell wall disruption (Figure 7) [76], which makes this electrotechnology a more effective cell disintegration technique than PEF. Moreover, air bubbles that are initially present in water or formed due to local heating will likewise be involved in, and accelerate the process [72,75,76].

Although the basis of the mechanism of all HVED extraction systems is the same, they can be divided into three general categories when considering the differences in the electrodes and the local electric field concentration modes: batch, continuous, and circulating extraction systems. In the batch system, a high-intensity electric field is concentrated at the needle electrode and discharges occur in the chamber. To reduce the extraction time, a continuous HVED extraction system with a continuous annular gap-type treatment chamber has been proposed, with the processing capacity significantly improved, and the clogging prevented. However, to increase processing capacity while maintaining higher yield, a circulating extraction system has been designed, with the electrodes composed of a needle and a ring [77].

Although on one hand, HVED as a green extraction technique has several advantages over conventional techniques, on the other hand, the non-selective extraction and release of all cellular material may create operational problems and increase the subsequent downstream purification costs. Moreover, the technological solutions demand for reliable high pulse power generators along with process limitations (such as operation only in batch mode) might represent the main factors hindering the industrial exploitation of this technique [78].

### 2.6. High-Pressure Homogenization (HPH)

**Figure 8:**
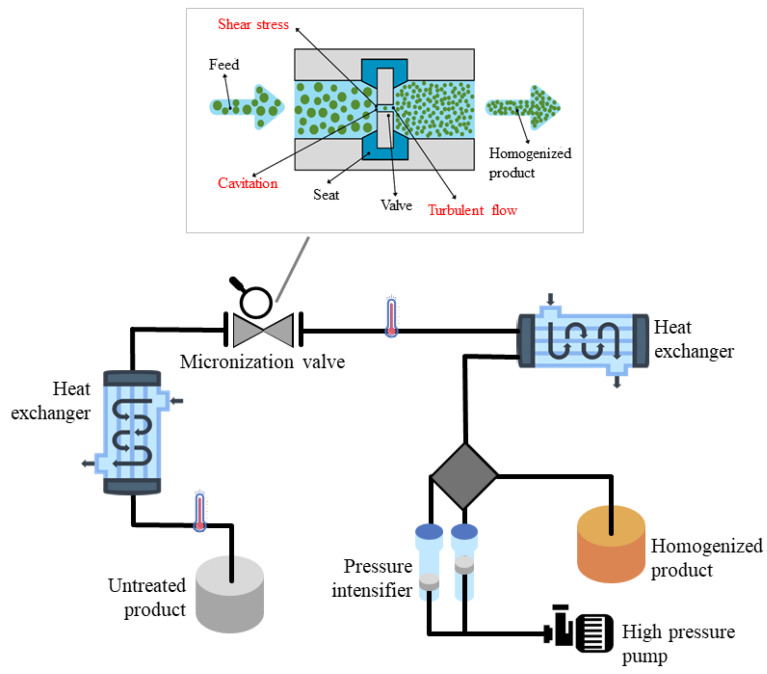
Schematics of the operating principle of a typical HPH system and focus on the homogenization valve.

The growing interest in environmental protection and food sustainability has attracted greater attention to alternative technologies such as high-pressure homogenization (HPH). It is a green technology with low energy consumption that does not generate high CO_2_ emissions or polluting effluents. In the homogenization process, the process fluid flows through the homogenization valve, where intense fluid mechanical forces are generated, which cause the particles suspended in the fluid to disintegrate (Figure 8). In the case of biomass, the particle disintegration corresponds to full cell disruption, with the associated release of intracellular material [28,79]. Moreover, also the total surface area of the newly formed particles increases, resulting in a significant improvement in the physical stability of the product. The fluid undergoes mechanical stress (shear, hydrodynamic, and cavitation effects) and a temperature increase (thermal effect) of about 2–3 °C for every 10 MPa of homogenization pressure [80]. The particle size decreases, and a more homogeneous distribution is obtained, facilitating operations such as mixing and emulsifying.

The existence of valves of different geometries has given rise to the design of equipment capable of working at pressures higher than 400 MPa. Therefore, three types of homogenizations are distinguished, according to the valve geometry:

- Standard homogenization for pressures between 0 and 50 MPa;
- High-pressure homogenization (HPH) for pressures between 50 and 300 MPa;
- Homogenization at very high pressure (UHPH) for pressures equal to or greater than 400 MPa.

The possibility of operating continuously for a great diversity of pumpable fluids has made it possible to extend the applications to the activation or inactivation of enzymes, reduction of the microbial load, processes of mixing, dispersion, emulsion, or encapsulation, processes of cell disruption, and protein modification. The use of HPH to valorize food residues has two objectives:

- improving the extraction capacity of intracellular structural components;
- improving the technological functionality of bioactive compounds.

**Figure 9:**
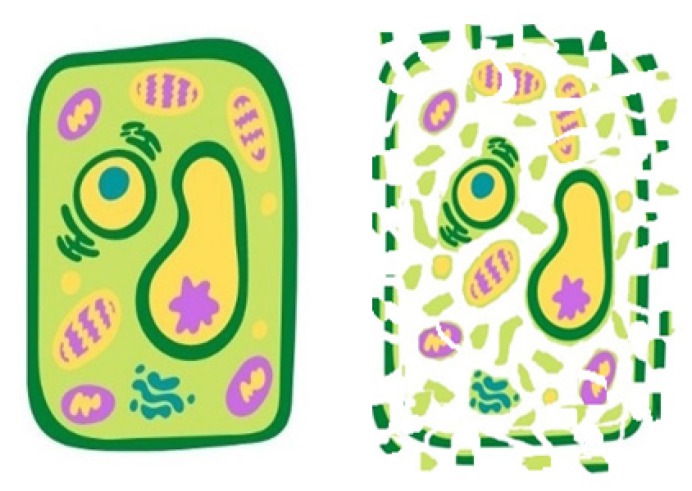
Mechanism of cell disintegration induced by HPH.

The application of HPH treatment leads to the destruction of plant tissues, cell walls, membranes, and organelles (Figure 9), improving the mass transfer of solvents into materials and recovering high-added value compounds [31].

The degree of cell destruction appears to be strongly dependent on the characteristics of the matrix and the intensity of the HPH in terms of working pressure and the number of passages through the homogenization valve [81].

The theory of homogenization is based on the flow-induced deformation of macromolecules.

During the passage through the valve, suspended particles undergo fluid dynamic stresses, leading to their rupture above a certain pressure. The sudden pressure drops at the valve exit induce sample acceleration and cavitation, leading to high kinetic energy, responsible for intensive collisions among particles and between particles and instrument walls [31].

## 3. Green Extraction Process: Synergism between Solvents and Technology

**Figure 10:**
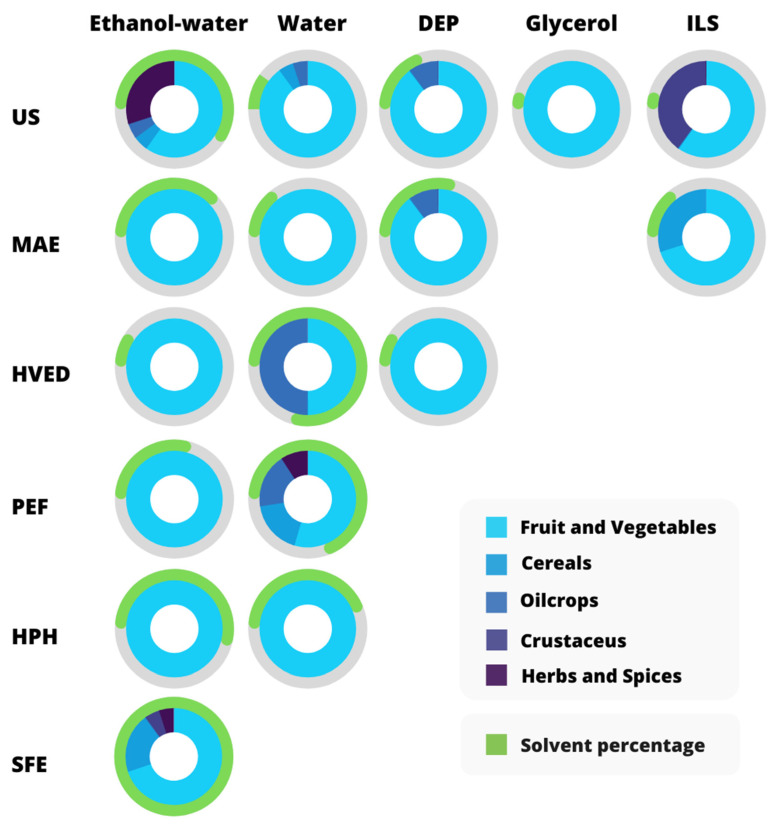
Classification of the green extraction technologies as a function of the solvent used and the food residues source.

The added value associated with the extraction of bioactive compounds from agri-food by-products is a crucial step for their minimization and environment-friendly valorization. In this context, approaching greener extraction alternatives using the above-mentioned emerging technologies and solvents with tunable properties, could be the desirable direction [82]. In the following section, a comprehensive and up-to-date analysis of the combined application of green solvents and emerging technologies in the extraction of antioxidant compounds from agri-food by-products is reported. Moreover, a classification of the used solvents and applied technologies, highlighting its affinity with the type of solvent, matrix, and target bioactive compounds is presented in Table 4, according to the main findings achieved in the last six years. Additionally, Figure 10 is intended to give a qualitative and immediate idea of Table 4, guiding the reader throughout it.

### 3.1. Neoteric Solvents

Neoteric solvents, characterized by an unconventional structure, are versatile as they can be used in a wide range of applications by varying their chemical constituents. The most attractive property of this class of solvents is their tunability [129]. Ionic liquids (ILs), deep eutectic solvents (DES) including Natural Deep Eutectic Solvents (NaDESs), and supercritical fluids belong to this category [1].

However, there are still some gaps to fill, namely, the production cost, availability, purity, safety regulations and toxicity, disposal, and recycling procedures.

In particular, the concern for their sustainability due to the high environmental persistence and the global heating potential (greenhouse gases) limits their wider application [130].

#### 3.1.1. Ionic Liquids

Within the neoteric solvents, ionic liquids (ILs) have attracted great interest as potential substitutes for conventional organic solvents in the extraction of bioactive compounds from natural sources [131,132,133]. They are a class of salts, composed of relatively voluminous organic cations and inorganic anions, where the asymmetry of the structure reduces the energy of the crystal lattice, thus lowering the melting point of the salt to values below 100 °C [134].

Thanks to high values of vaporization enthalpies (ΔH_vap_), they are non-volatile, have negligible vapor pressure, high chemical and thermal stability, and considerable solvating power [135]. Although their use does not cause any air pollution, to date, several used ILs have shown potential environmental risks and could pose serious threats to aquatic and terrestrial ecosystems after their release [136], also due to the numerous steps required to produce them, including a range of toxic and harmful intermediates [1]. Moreover, one of the drawbacks associated with these green solvents, in comparison with conventional solvents, is their high density and viscosity [137]. The latter can be lowered by temperature, which is an important factor in the mass transfer process [138].

As for their use, since ILs are immiscible in water and soluble in organic species, they are suitable solvents for the extraction of a variety of bioactive compounds [41]. ILs have been applied also to extract antioxidants from agri-food by-products, such as gamma-oryzanol from rice husk [138], tyrosol from oil mill wastewater [41], naringin from pomelo peels [84], and carotenoids from shrimp shell [50] and orange peels [86]. Furthermore, the extraction of bioactive compounds from agri-food by-products using ILs combined with innovative extraction technologies has gained relevance in the last years, pursuing the principles of green chemistry. Research in this field is mainly based on MAE technology, because of the possibility to achieve high extraction yields in a short time [131]. Ionic liquids are considered good microwave absorbers since heated rapidly and uniformly under microwave irradiation [15]. Thus, the synergistic use of ILs and MAE could contribute to reducing extraction time and energy consumption, and is, therefore, considered as a green extraction approach to extract different compounds such as phenolics, essential oils, alkaloids [131].

Extraction time was shown to be reduced by 92% when comparing traditional methods, such as heat reflux extraction, with IL-based MAE for naringin recovery from pomelo peels [84]. UAE could also be used as an alternative to MAE at limited operation scales, for extracting thermolabile compounds, since UAE could enhance mass transfer mechanically with no need for heating [131]. However, the major issues preventing the industrial implementation of ionic liquids are still not fully solved. Ensuring recyclability and reusability will be critical for successful industrial implementation. Unknown toxicity is often a major criticism of ionic liquids [1]. However, it should be also considered that, since a wide range of ILs can be obtained by changing the cation and/or anion, it is possible to select less toxic ILs for the required extraction application. The tailor-made physicochemical properties of ILs may counteract the negative concerns, providing new challenges to research in the field [131].

#### 3.1.2. Deep Eutectic Solvent (DES)

Solvents known as DESs including NaDESs, composed of plant metabolites [1], have been defined as sustainable solvents as substitutes of conventional organic solvents to extract bioactive compounds, thanks to their low toxicity, biocompatibility, and recyclability [8].

The main concept is based on the combination of a hydrogen bond acceptor (HBA) and a hydrogen bond donor (HBD) mixed at a suitable temperature [139]. These hydrogen bond interactions determine the formation of eutectic mixtures, characterized by a melting point lower than that of the single constituents [140]. The most common HBA component is choline chloride (ChCl), an inexpensive and non-toxic salt, while the most widely used HBDs are urea, ethylene glycol, glycerol, but also alcohols, amino acids, carboxylic acids, and sugars [141,142].

Compared to common organic solvents, DESs offer many advantages such as low price, simple preparation, and easy availability. Compared to ILs, they show similar characteristics such as low vapor pressure and low melting points, non-flammability, dipolar nature, chemical, and thermal stability, high solubility. In addition, they can compensate for the drawbacks of ILs such as toxicity (DESs are ten times less toxic than ILs), non-biodegradability [143,144], complex synthesis requiring purification, and the high initial cost of raw materials [145]. Moreover, the DESs and NaDESs have also been shown to have high potential as solvents when combined with innovative extraction technologies, such as HVED, MAE, and UAE [146]. The combination of these solvents with green extraction techniques also allows the improvement of extraction yields, which is attributed to the heat generated by ultrasounds and microwaves decreasing the solvent viscosity and enhancing the penetration of DESs and NaDESs into the biomass matrix [147]. Viscosity is often a problem for many neoteric solvents that have detrimental effects on mass transfer, reaction rates, and handling.

The usage of DESs and NaDESs alone and combined with emerging technologies to recover antioxidant compounds from agri-food by-products (namely, winemaking, olive oil, tomato, pear, onion processing by-products) have shown promising results. The ability of DES to donate and accept protons and electrons as well as to form hydrogen bonds gives them good dissolution properties towards phenolic compounds, as recently explored. For instance, El Kantar et al. (2019) investigated the efficiency of DES synthesized with lactic acid and glucose coupled with HVED as pretreatment, to improve the extraction kinetics, for the recovery of polyphenols, specifically naringin, from grapefruit peels. The extraction of naringin improved by 3 times than when employing conventional extraction using ethanol [88]. Also, Pal and Jadeja (2019) proposed the use of DES combined with MAE to improve the extraction efficiency of antioxidants from onion peels [92]. The authors demonstrated a recovery of bioactive compounds approximately 1.5 times higher when compared to soxhlet extraction using 70% aqueous methanol, and a 24-fold reduction in extraction time. Additionally, the synergistic irradiation effect of MAE and UAE (UMAE) was studied by Panic and co-workers. This double irradiation resulted in doubled extraction efficiency. Sonication contributed to the mechanical cell rupture, while microwaves promoted the release of the intracellular target compounds [89].

However, for an industrial application of these solvents, their recycling is one of the main challenges to overcome to conduct an efficient process [146]. The low vapor pressure of eutectic mixtures is considered a drawback for its recovery. To this purpose, recently, Panić et al. (2019) used an adsorption chromatography with macroporous resin to recover up to 70.36% of the anthocyanins extracted from grape pomace with the solvent recycling yield of 94.78% [89]. Therefore, key factors involved in the recovery and recycling of DESs or NaDESs should be considered to implement the use of these solvents at an industrial scale. In this sense, an interesting approach is to directly use the whole extract, after having evaluated the toxicity profile, which would lead to a more sustainable extraction process, reducing the costs associated with downstream steps [8].

### 3.2. Supercritical Fluids (SCFs)

Extraction with supercritical fluids represents a valid alternative to conventional solvent extraction systems. Supercritical fluids (SCF) are substances for which both pressure and temperature are higher than their critical values assuming physical characteristics intermediate between those of a liquid and those of a gas [148,149]. Therefore, the synergism among density, low viscosity, diffusivity, near-zero surface tension, pressure, and temperature dependence allow supercritical fluids to easily penetrate a microporous matrix to extract intracellular compounds [150].

The most widely used supercritical fluids are water, carbon dioxide, helium, refrigerants, and hydrocarbons, but the health and safety benefits are particularly evident in the use of supercritical CO_2_ and supercritical water. However, the broader applicability of supercritical fluids extraction, particularly with regards to its industrial exploitation, remains quite controversial, due to its higher energy consumption, risk of hydrolysis, degradation reactions, higher CO_2_ emissions, and capital costs [151].

#### 3.2.1. Water

Water as an extraction solvent can be used either at supercritical or subcritical conditions. Supercritical water (SCW) can be used as a solvent in many reactions and is considered the cleanest solvent [152]. It exists at temperatures above 374 °C and pressures above 22.1 MPa.

It is characterized by a much lower dielectric constant and fewer hydrogen bonds and with less persistence than liquid water, which makes it to be considered a non-polar solvent [152].

The oxidation of waste in supercritical water (SCW) has been investigated for the valorization of food residues. However, one of the problems related to the use of supercritical water is corrosion, which, even today, is being deeply studied to obtain a satisfactory application on an industrial scale [153]. In this sense, the use of subcritical water extraction (SWE) or pressurized hot water extraction (PHWE) as a “green” extraction solvent has progressively spread in the last years [154]. It is localized in the region of the condensed phase of water between the temperature range from 100 °C to 374 °C. Various reports have shown that at a certain temperature and applied pressure, the polarity of water can be varied close to that of alcohols. In the case of PHWE, the density of the water remains almost constant so that the effect of pressure on the properties of the water is minimal [153]. The pressure is usually varied from 10 to 80 bar to keep the water in its liquid phase. The effect of the use of subcritical water on the extraction of phenolic antioxidants from fruit, vegetables, and herb by-products such as peach palm residues [155], kiwifruit peels and pomace [156,157], onion skin [158], pistachio hulls [45], sage by-products [48], papaya seeds [22] and date residues [33] has been recently investigated. These studies demonstrated that subcritical water showed significantly higher extraction yields and antioxidant activities in comparison with traditional solvent extraction. In particular, the total phenolic compounds from peach palm by-products were 2.5-fold higher than the values obtained from conventional extraction [155] and the concentration of gallic acid in the extract from pistachio hulls was 13.2-fold higher than that in the aqueous methanol extracts [45]. Moreover, the use of subcritical water combined with 30% of NADEs led to a significant increment of extracted phenolic compounds (catechin and epicatechin 45.05 and 47.98%, respectively) from winery by-products in comparison with the use of subcritical water alone [159].

#### 3.2.2. Carbon Dioxide

Supercritical CO_2_ is non-flammable, inert, non-toxic, has a relatively low cost, and has moderate critical constants. The value of the dielectric constant identifies it as a non-polar substance and its solvation strength can be fine-tuned by adjusting the density of the fluid, which significantly increases in the supercritical region. CO_2_ leaves fewer residues in products than conventional solvents and is available in relatively pure form and large quantities.

Supercritical CO_2_ allows the extraction of thermosensitive compounds, sensitive to oxidation and low molecular weight, such as polyunsaturated fatty acids (ω3, ω6), vitamins, cannabinoids, flavonoids, carotenoids, sterols, tocopherols. However, although it is mainly used to extract poorly polar compounds, the addition of co-solvents showed to be a valid approach in extracting more polar compounds. Ethanol was selected as the best co-solvent because of its suitability for food applications [160]. Indeed, the extraction from grape pomace with the use of supercritical mixtures of CO_2_ and water/ethanol (57% *v*/*v*) as co-solvent was shown to give yields of 71.32 mg GAE (Gallic Acid Equivalents) per g of dry weight enriched in flavanols [96]. The increase of ethanol concentration added within (5–10%) increased the final yield and significantly decreased the extraction time from 100 to 58 min [103]. However, higher ethanol concentrations could hinder the extraction of the target compounds, since the hydrogen from the ethanol molecule may form hydrogen bonds with the oxygen of other molecules, increasing the required energy to separate ethanol molecules, causing a decrease in the process yield [103].

Despite this technique allows selective extraction and great versatility, it has limiting factors linked to high processing costs and complex industrial equipment, in comparison with conventional processes. Thus, the application of other technologies such as ultrasounds, combined with SFE, may be useful in addressing these limitations and enhancing the extraction yield of target compounds from food residues. Santos-Zea et al. (2019), demonstrated that the synergistic effect of the application of ultrasound on the SFE of bioactive compounds from agave bagasse led to an increase in the antioxidant activity of the extracts (42%) [101].

### 3.3. Supramolecular Solvents (SUPRAS)

SUPRAS are nanostructured liquids produced in colloidal solutions of amphiphilic compounds through two sequential self-assembly processes, which take place respectively at the molecular and nanometric level and are excellent solute extractors in analytical processes, thanks to the high concentrations of amphiphiles [161].

Unlike organic solvents and ionic liquids, SUPRAS components arrange themselves in orderly structures, which confer their exceptional properties. They are characterized by remarkable properties for the solubilization of a series of polar and non-polar compounds and can be customized and produced spontaneously at reduced costs, using a minimal amount of solvent [162]. Furthermore, SUPRAS are non-volatile, non-flammable, and the ordered structure can be adjusted by the correct selection of the amphiphilic molecules and the environment to meet specific functions [162].

SUPRAS have been recently applied for the valorization of coffee residues to recover caffeine, and antioxidant compounds [106]. Extraction yields with SUPRAS demonstrated to be significantly higher than those obtained with organic solvents usually employed in the recovery of valuable compounds from coffee wastewater [104], spent coffee grounds [106], and coffee pulp [105]. Additionally, SUPRAS extraction improved most of the coffee wastewater quality parameters, simply by mixing them with the wastewater. The phase separation occurred spontaneously due to the different densities [104]. Moreover, ultrasonication was found to be an effective strategy to control the self-assembly of the amphiphilic copolymer into nanoparticles and its interaction with curcumin in the field of drug-delivery systems [161].

### 3.4. Bio-Based Solvents

The chemical industry is now considering renewable sources as the primary source of sustainable solvents [1]. Bio-based solvents are defined as solvents produced from renewable biomass sources such as wood, starch, vegetable oils, or fruits [163]. They are produced in a biorefinery that aims at maximum recovery and production of products with high-added value. Despite their great potential, the scale of biorefineries is still mainly limited to pilot or laboratory scale plants [164], since the sustainability of biomass production highly depends on the implementation of land-management practices [1].

These bio-solvents have a high solvent power, are non-toxic, non-flammable, and biodegradable. Their drawbacks are related to high viscosity and boiling point, high cost, and generation of off-flavors [165].

Ethanol is the most common bio-solvent, obtained from the fermentation of sugar-rich materials, such as sugar beet and cereals. Despite its flammability and potential explosivity, ethanol is used on a large scale thanks to its availability at high purity, cheapness, and biodegradability. Among the bio-solvents, there are also terpenes, such as d-limonene extracted from citrus fruit. Because of its low polarity and its solvent power, d-limonene is largely used for the extraction of fat and oils [166]. In addition, glycerol, a by-product from the trans-esterification of vegetable oils, is very common in the cosmetic industry as a solvent for the maceration of herbs and spices [165].

#### 3.4.1. Ethanol

The most used green solvent for extraction processes is bioethanol, produced through the anaerobic fermentation of sugars [10].

To obtain a high percentage of polyphenol recovery, ethanol is always combined with an adequate amount of water. However, the final composition of the solvent is defined by the nature of the solute. For example, an aqueous solution of 80% (*v*/*v*) ethanol was found to be very effective for pomace of various red grape varieties, providing total polyphenol yields of 69.3–131.7 mg of gallic acid per g of dry weight [167]. For the extraction of polyphenols from seeds, 50% (*v*/*v*) of ethanol was much more effective than water and aqueous acetone [168]. Ethanol-water solutions showed higher efficiency in extracting antioxidant compounds than a mono-component solvent system (water, pure ethanol) because the mixture, inferring the change in the polarity of the compounds, reaches the polarity of diverse compounds [113,169]. Many authors proved the strong influence of the interaction between emerging technologies and the hydroethanolic mixture composition on the extraction efficiency of bioactive compounds from agri-food by-products. A 50% (*v*/*v*) ethanol solvent presented the best extraction yields for anthocyanins, polyphenols, flavonoids, and tannins from jabuticaba peels, demonstrating its synergistic effect with the ultrasounds at an intensity of 3.7 W/cm^2^ [113].

A comparison between the application of MAE and UAE aimed at the valorization of peach juice residue, using 70% (*v*/*v*) ethanol, was conducted, providing a preliminary estimation of the potential economic and environmental impact of these technologies [111]. The authors proved that MAE was more efficient in extracting the vitamin C with half extraction time compared to UAE. Additionally, MAE would also be less impactful than UAE in terms of greenhouse gas emission and energy requirements [111]. However, the effect of the combination of PEF pre-treatment to achieve cell permeabilization and the subsequent UAE with 55% (*v*/*v*) ethanol on the extractability of bioactive compounds from fresh rosemary and thyme by-products demonstrated to be the most efficient solution that is attracting considerable interest [51]. PEF pre-treatment enhanced the antioxidant capacity of rosemary extracts resulting in a 1.3-fold increase, when compared to UAE individually applied [51]. Additionally, the effect of the cell tissue disruption of ultrasounds on lettuce waste hydro-alcoholic dispersions (50–75% (*v*/*v*) ethanol) was also compared to the one associated with the mechanisms involved in the HPH treatment [31]. Different from UAE, which induced progressive destruction of cellular structures (vacuoles), HPH-induced disruption was not gradual, so that lower energy was required to obtain higher tissue disruption. Although HPH promoted a much more intense cellular rupture, as a pre-treatment to UAE, it resulted in lower phenolic yields (25%) as compared to UAE. These results could be possibly be explained by the decompartmentalizing effect of HPH on the oxidative enzymes entrapped in the plant matrix, while US did not cause changes in the polyphenol oxidase (PPO) conformation [170].

#### 3.4.2. Glycerol and D-limonene

Glycerol is a major by-product of biodiesel production and fatty acid manufacturing via the hydrolysis of triglycerides. However, as a by-product, crude glycerol requires further purification if it is intended to be used as a solvent [1].

The glycerol molecule has various advantages as it is soluble in water and methanol, slightly soluble in other polar solvents, and insoluble in non-polar solvents. Glycerol is a cheaper bio-solvent than ethanol and has no toxicity as it is a natural constituent of food. It is highly viscous and may therefore be problematic for many chemical processes. However, it could be used together with a cosolvent to lower its viscosity (e.g., DES, water, ethanol) [11]. Several studies highlighted glycerol as a bio-solvent with an important prospect in the extraction of polyphenols [98]. The effects of glycerol on the extraction yields of phenolic compounds from grape pomace and potato peels have also been investigated in combination with emerging extraction technologies, as UAE, to exploit their synergistic contributions regarding both the improvement in the mass transfer and the disruptive effect on the vegetable cell tissue associated to the technology [118,119].

Among the terpenes, a growing interest has emerged in D-limonene in recent years. It is a constituent of the essential oils resulting from citrus fruit by-products and is considered as GRAS (Generally Recognized as Safe) by the FDA. D-limonene was used as an alternative solvent to substitute n-hexane in the extraction of bioactive natural products (carotenoids, oils, and aromas) [4]. Limonene was also effectively applied for the extraction of fat-soluble vitamins from anchovy fillet leftovers [171].

#### 3.4.3. Water

Water can be considered as a potentially green solvent since it is non-toxic to health, it has a low environmental impact, it is low cost in terms of production, transportation, and disposal. In addition, the capability of this solvent to tune its properties by changing the conditions (e.g., temperature, pressure) has contributed to the increasing interest in using water as an extraction solvent [1].

The use of water as a green extraction solvent has been widely combined with the application of emerging extraction technologies, to enhance the extractability of antioxidant intracellular compounds from agri-food by-products. In particular, several authors have compared the effect of different technologies, namely PEF, HVED, and UAE, on the improvement of the extraction yields of bioactive compounds from agri-food peels and pomace [44,62,125], according to the cell rupture mechanisms related to each technology, to the plant matrix and tissue (stems, seeds, peels, pomace), to the physical and chemical properties of the target compounds (solubility, polarity).

HVED treatment resulted to be more efficient in terms of energy input than PEF to achieve a higher cell permeabilization degree and to improve phenolic compounds aqueous extraction from mango peels [125]. These results could be related to the ability of HVED to induce fragmentation of the cell tissue due to the propagation of shock waves and the explosion of cavitation bubbles [76]. However, the extracts obtained by HVED were less clear and stable than the extracts obtained by PEF, which is characterized by a better selectivity related to the electroporation phenomenon, facilitating the subsequent separation and purification processes [125]. These results were in accordance with the main findings of Barba et al. (2015), who proved that HVED was the best technology to achieve the highest phenolic compounds recovery from grape pomace with lower energy requirement than PEF and ultrasound (US) [62]. However, HVED was less selective than PEF and US regarding the anthocyanins recovered, because of the different mechanisms involved. HVED was able to induce the release of cell-wall-linked phenolic compounds and particularly proanthocyanidins, which may interact with polysaccharides. However, US and PEF were more efficient than HVED in promoting localized fractures in the inner layer of the epidermis, where vacuolar anthocyanins were located.

Given these considerations, the most promising application of HVED could be for enhancing the extraction of oil and phenolic compounds from seeds, with hard and lignocellulosic tissue, due to the cell disruptive mechanisms involved in the process [44].

Another technology, based on cell tissue disruptive mechanism, is high-pressure homogenization (HPH), used as a mechanical disruption method to recover valuable compounds from agri-food by-products, aiming at their total valorization, using water as solvent [172]. This technique is mainly applied as a wet milling technique, to improve the rheological behavior of the suspensions controlling the particle size distribution of the residues [79,173]. HPH-induced micronization of tomato peels suspensions promoted the complete disruption of the plant cells. HPH enabled the increased release of intracellular compounds (proteins 70.5%), polyphenols (32.2%)), and the recovery of up to 56.1% of the initial lycopene content [28].

A growing interest has recently focused on microwave irradiation based on the hydrodiffusion phenomenon that exploits in situ the water present in the plant tissue. For example, microwave irradiation induced the heating of the internal water within the plant tissue of grape by-products, allowing the destruction of the cell tissue and the release of the intracellular bioactive compounds [57], by breaking down ester- and glycoside-bound phenolic compounds [67].

An interesting example of a sustainable and integrated strategy is the extraction of essential oil and polyphenols from orange peel using microwave and ultrasound technology exploiting only in situ water. Microwaves caused the evaporation of interstitial water and induced the release of the essential oil. Essential oil–vapor mixture was then condensed, the essential oil collected, while water was recycled and used for the polyphenols extraction from the MAE residues, performed by UAE [23]. This extraction process enabled the obtainment of high-added value compounds in a shorter time than conventional extraction, through a closed cycle using only water provided by the plant [23].

## 4. Green Solvents Selection

### 4.1. Physical Properties

Recently, the solubilization power of solvents can be theoretically predicted using computational programs, playing a key role in guiding the screening and selection of solvents in various applications [174]. Several of them are simple models predicted using physical properties of solvents like Kauri-butanol index, Kamlet-Taft scale, Hildebrand solubility parameters. Currently, two more powerful 3D-space computational programs, i.e., the Hansen Solubility Parameters (HSPs) and a COnductor like Screening MOdel for Realistic Solvents (COSMO-RS), are being used to predict the most suitable solvents for the extraction of natural products [175,176]. The solubility theory approach, which is based on the principle “like dissolves like”, can be helpful in giving a first approximation of the most suitable solvent for a given extraction process, thus avoiding numerous experiments and useless experimental conditions [3]. Table 5 summarizes the properties measured by the above-mentioned computational methods, together with the associated advantages and limitations.

In particular, HSPs are based on the concept that the total cohesive energy density is approximated by the sum of the energy densities required to overcome atomic dispersion forces (δd2), molecular polar forces related to dipole moments (δp2) and hydrogen-bonds between molecules (δh2) [3,88,176]. Moreover, for HSP solvent optimization, a composite affinity parameter, the relative energy difference (RED), is given by the ratio of Ra (the distance of a solvent from the center of the Hansen solubility sphere) and Ro (the radius of a Hansen solubility sphere), to determine the solubility between solvent and solute. Thus, this parameter is able to distinguish good potential solvents (RED < 1, the studied solvent is within the solubility sphere and should dissolve the target compound [177]), medium solvents (1 < RED < 3), and poor solvents (RED > 3) [3,176]. However, recently, COSMO-RS has proven to be quite powerful, and it may currently represent the best link between the world of chemical quantum mechanics and engineering thermodynamics. In particular, COSMO-RS consists of a two-step procedure: in the first step, the COSMO model is applied to simulate a virtual conductor environment for the molecule of interest (microscopic approach). The second step uses the statistical thermodynamics calculation (macroscopic approach). In such kind of environment, the molecule induces a polarization charge density σ on the interface between the molecule and the conductor. This polarization charge density is used for the quantification of the interaction energy of pair-wise interacting surface segments [182]. The 3D distribution of the polarization charges σ on the surface of each molecule is converted into a surface composition function (σ-profiles) that provides information about the molecular polarity distribution and is accordingly integrated to calculate the chemical potential of the surface (σ-potential) [182,183]. The σ-profiles and σ-potentials, together with other thermodynamic parameters, can assure pre-screening and classification of various solvents [184].

Recent studies included the prediction of solubilization of several compounds with high antioxidant activity including polyphenols, limonene, and alpha-mangostin in various “green” solvents from agri-food by-products, as orange [185] and grape peels [88], and mangosteen pericarps [176], respectively. These predicted results were successfully compared with experimental data, demonstrating their successful application in the extractions of natural compounds for the replacement of conventional solvents with “green” ones, also aided with emerging extraction technologies. In this framework, El Kantar et al. (2019) studied the application of HSPs as a predictive method to evaluate the solubility of polyphenols from grape peels in aqueous glycerol, using HVED as pre-treatment electrotechnology, to improve their extractability. Results showed a reduction of 6 times in the HVED energy input, when using 20% (*w*/*v*) aqueous glycerol instead of conventional solvents and demonstrated the ability of the theoretical method to predict the solubilization of naringin, reducing the number of experiments [88]. COSMO-RS was also used to determine the ability of bio-based solvents chloropinane and chloromenthene, from pinene and limonene, to solubilize β-carotene, vanillin, and rosmarinic acid when using n-hexane [182]. Chloropinane and chloromenthene were 3.5 and 2 times more efficient than hexane for rosmarinic acid solubilization, and β-carotene and vanillin were 6 and 20 times more soluble in chloropinane than in hexane.

HSPs and COSMO-RS applications have also been compared for the extraction of alpha-mangostin from mangosteen pericarps using green solvents as an alternative to petroleum-based dichloromethane. COSMO-RS showed a higher consistency with the obtained experimental data than HSPs [176]. COSMO-RS has been shown as the most accurate method for solvent solubility screening of “green” solvents.

### 4.2. Environmental Assessment

Although water is widely used as a solvent since it is the cheapest and the most available solvent for “green” extractions, it cannot be truly considered as a “green” solvent. It is highly evaporative and consequently has high energy consumption, CO_2_ emissions, and, thus, unfavorable environmental impact [1]. In addition, terpenes such as α-pinene, and d-limonene have recently been identified as high-risk solvents due to environmental emissions. Their moderate inhalation toxicities and high photochemical ozone creation potentials (POCPs) are made worse by their environmental partitioning into the air [1,186]. Therefore, sustainable development pays attention to considering the technical, economic, and environmental impacts of solvents throughout their lifetimes, which could be detrimental when applied at an industrial scale. In this sense, the Life Cycle Assessment tool can help to balance production, application, and disposal, while accounting for environmental impact [1].

Khoo et al. performed a detailed LCA to produce bioderived 2-MeTHF from three biomass sources (corn stover, sugar cane bagasse, and rice straw). The LCA demonstrated that the energy usage and environmental damage caused by crop production far outweighed that of biomass processing and how solvent sustainability is also dependent on the cultivation step [187]. Therefore, it is important to highlight that an integrated development of computational methods (HSP or COSMO-RS) in combination with LCA, promotes the application of “green” solvents in extraction processes. Jin et al. have developed a 10-step method that encompasses a hierarchy of assessments, including the study of the physical properties, the techno-economic and environmental assessment, designed to help guide the development of new biobased solvents and identify the “hotspots” of green extraction processes [1,188].

## 5. Conclusions

Nowadays, the food industry is constantly facing the continuous evolution of consumers’ needs, progressively interested in food products with all-natural ingredients and the use of green processes. In this sense, the valorization of agri-food by-products, as a natural and cheap source, through the recovery of valuable intracellular compounds, including antioxidants and phenolic compounds, could represent a useful and sustainable strategy to deal with these challenges. In this framework, the application of green technologies, such as Supercritical Fluid Extraction (SFE), Microwave-Assisted Extraction (MAE), Ultrasound-Assisted Extraction (UAE), High-Pressure Homogenization (HPH), Pulsed Electric Fields (PEF), High Voltage Electrical Discharges (HVED), able to induce the partial or total disintegration of the cell envelope, which constitutes a physical barrier to the diffusion of the bioactive compounds, represents an emerging approach to intensify their extractability. In addition, these technologies enable the reduction of solvent consumption, time and energy requirements, and the improvement of the extraction yields, leading to greener processes than through conventional extraction techniques.

The extraction process constitutes a critical issue for the valorization of agri-food by-products since it depends not only on the source composition and the tissue considered (peels, stems, seeds, shell) but also on the physical and chemical properties of the desired compounds. Therefore, the selection of the most appropriate extraction technique should rely on the nature of the matrix and the localization of the target compound inside the cell, which the different cell rupture mechanisms associated with each technique depend on. Additionally, solvent selection represents another important aspect that contributes to making an extraction process sustainable. The use of the most suitable, biocompatible solvents, primarily ethanol and water, coupled with environmentally friendly technologies, represents an integrated approach towards the development of “green” extraction processes.

Future research should address not only the scientific aspects of the integration of the novel technologies but also cost minimization, especially in terms of investment costs, which currently limits their implementation at larger scales, as well as the sustainable valorization of agri-food by-products, driven by an environmentally and socially friendly perspective. The expected long-term goal is represented by the industrial implementation of the emerging technologies in a biorefinery approach for the green extraction, with high efficiency and low-costs and using agri-food by-products as cost-effective sources of natural compounds for the commercialization of naturally-derived and high value-added products, such as food additives, contributing to meet consumers’ increasing demand for cleaner labels.

## Figures and Tables

**Table 1:** Green solvents categories with their respective advantages and disadvantages.

Classification	Advantages	Disadvantages
**Neoteric solvents**	Extraction of triglycerides, natural colorants, aromas, polyphenols	
Ionic liquids (ILs) [6]	Negligible vapor pressure Thermally stable at temperatures >200 °C Exceptional solubility for organic, inorganic, and organometallic substances	Medium-high viscosity values Some are expensive Toxicity issues not fully addressed
Deep Eutectic Solvent (DES) [7,8]	Ease of preparation Excellent solubilization capacity of diverse compounds with poorly water solubility Low cost High biodegradability Adjustable viscosity	High viscosity compared to many conventional organic solvents Toxicity issues not fully addressed
**Supercritical fluids (SCFs)**	Decaffeination of tea and coffee, extraction of lecithin from oil	
Supercritical water	Renewability No toxicity issues	High energy requirements in the separation and reuse processes Equipment oxidation issues
Supercritical carbon dioxide [9]	Inexpensive No risks associated with the use of organic solvents Odorless, non-toxic, renewable Simple industrial recycling	High pressure required Poor ability to dissolve polar and ionic species High equipment maintenance costs
**Bio-based solvents**	Extraction of pigments, antioxidants	
Ethanol [10]	Appreciable solubility of organic compounds in the supercritical state Ease of recovery	Net increase in emissions Flammable and potentially explosive Corrosive in nature
Glycerol [11]	Extraction of polyphenols Colorless, odorless, sweet-tasting product and biodegradable Chemical stability during storage High boiling solvent	High operating and investment costs
Terpenes [4]	Extraction from fats and oils Ease of recovery and reuse Biodegradability Non-flammability	Low polarity High volatility
**Supramolecular solvents (SUPRAs)** [12]	Extraction of alkaloids, bioactive compounds, removal of pesticides, surfactants, dyes	
**-**	Capability to extract amphiphilic compounds	Extraction of solutes from solid samples not deeply explored

**Table 2:** Classification of the main agri-food by-products, the antioxidant compounds they are rich in, and the related expected beneficial properties.

Source	Main Antioxidant Compounds	Expected Health-Beneficial Properties	References
Fruit & Vegetables			
Grape pomace (peels, seeds, pulp, stems)	Flavonoids (anthocyanins, monomeric catechin, epicatechin), stilbenes (resveratrol), tannins, gallic acid	Oxidative stress, cancer, and disease risk reduction, cholesterol regulation	[15]
Peach residues (peels, seeds, pulp)	Phenolic compounds, carotenoids, vitamin C	Antioxidant, anti-hyperglycemic, anti-aging properties	[16,17]
Pineapple residues (peels, stem)	Phenolic compounds, proteolytic enzymes (bromelain), vitamins, carotenoids	Cytotoxic, antidiabetic, antihyperlipidemic, antioxidant properties	[18]
Banana peels	Phenols and flavonoids	Inhibition against diverse bacteria and fungi, and some cancer cells, blood sugar, and cholesterol reduction	[19]
Mango peels	Carotenoids, flavonoids, phenolic compounds, and vitamins	Reduction of the risk of cancer and coronary heart disease	[20]
Pomelo peels	Flavonoids (naringin, quercetin, rutin), vitamins	Antioxidant, anticancer, anti-inflammatory properties, lowering levels of blood cholesterol	[21]
Papaya seeds	Tocopherols, carotenoids, flavonoids tannins, fatty acids	Antioxidant properties	[22]
Orange peels	Flavonoids (hesperidin, narirutin), carotenoids, xanthophylls	Antioxidant activities, reduction in the incidence of cancer, heart disease, osteoarthritis, ocular disorders	[23]
Pomegranate peels	Phenolic compounds (punicalagin)	Antioxidant, anti-inflammatory, hepatoprotective, and antigenotoxic effects	[24]
Apple pomace	Phenolic acids (chlorogenic acid), flavonoids (catechins, epicatechins), dihydrochalcone (phloridzin)	Anticancer, anti-inflammatory, antibacterial, and antiviral properties	[25]
Chestnut by-products	Vitamin E, phenolic acids, tannins	Antioxidant, anti-inflammatory, and antimicrobial properties	[26]
Spent coffee grounds	Phenolic compounds (chlorogenic acid, hydroxyhydroquinone), flavonoids	Antioxidant, anti-inflammatory, anti-microbial, and cholesterol-lowering effects, prevention of degenerative diseases	[27]
Tomato pomace	Flavonoids and carotenoids (lycopene)	Reduction of the risk of cardiovascular diseases, atherosclerosis, prostate cancer	[28,29]
Artichoke wastes	Phenolic compounds (chlorogenic acid)	Scavenging capacities against reactive oxygen species and reactive nitrogen species, anti-obesity effects	[30]
Lettuce waste	Phenolic compounds (chicoric acid; luteolin-7-O-glucuronide)	Antioxidant properties	[31]
Carrot pomace	Carotenoids (β-carotene)	Antioxidant, anti-inflammatory properties, improvement of immune response	[32]
Onion peels	Flavonoids (quercetin and kaempferol)	Anti-inflammatory and anti-cancer effects	[33]
Potato peels	Polyphenols, phenolic acids (caffeic acid, syringic acid)	LDL-lipoprotein oxidation, prevention of platelet aggregation, and red blood cell damage	[34]
Eggplant peels	Phenolic compounds, ascorbic acid, anthocyanins (tulipanin, nasunin)	Antioxidant properties	[35]
Mushroom stalks	Ergosterol	Antioxidant properties	[36]
Cereals			
Rice bran	Tocopherols, tocotrienols, γ-oryzanol, tannins	Antioxidant, antihypertensive, antimicrobial, antidiabetic, anticancer properties, cholesterol-reducing effect	[37]
Wheat, barley, millet, sorghum	Phenolic acids, vitamins, minerals		[38]
Brewer’s spent grain	Minerals, vitamins, polyphenols, arabinoxylan, β-glucan	Enhanced glycaemic control, cholesterol-lowering effect, prebiotics effect, immunomodulatory activity, increased minerals absorption	[39]
Buckwheat sprouts	Flavonoids (rutin, quercetin), vitamins	Hypocholesterolemic, hypotriglyceridemic, anti-inflammatory properties	[40]
Oil crops			
Olive mill wastewater	Tyrosol, hydroxytyrosol	Prevention of Parkinson’s disease, hyperglycemia, cerebral ischemia	[41]
Olive pomace	Phenolic compounds, secoiridoids	Antioxidant and anti-inflammatory properties	[42,43]
Sesame cake	Polyphenols, lignan glucosides	Prevention of obesity and hyperglycemia, reduction of cholesterol levels	[44]
Pistachio hulls	Phenolic acids (gallic acid), gallotannins, flavonoids (quercetin, myricetin glycosides)	Prevention of cardiovascular disease, diabetes, high cholesterol levels	[45]
Herbs and spices			
Wild thyme by-product	Flavonoids, phenolic acids, essential oils (thymol)	Antimicrobial, antioxidant, anti-aging, anti-inflammatory, immunomodulatory and anti-cancer, liver protective, gastroprotective activities	[46]
Rosemary by-products	Polyphenols (rosmarinic acid, carnosolic acid, carnosol), essential oils		[47]
Sage by-products			[48]
Tea by-products	Phenolic compounds (chlorogenic acid), flavonoids (apigenin, luteolin), essential oils		[49]
Fish by-products			
Shrimp waste	Carotenoids (astaxanthin)	Antioxidant activity, inhibition of lipid peroxidation	[50]

**Table 3:** Advantages and disadvantages of green extraction technologies.

Extraction Method	Advantages	Disadvantages
SFE	High extraction yields, fast extraction, automated system, no filtration required, possibility to reuse CO_2,_ no use of toxic solvents, possibility to tune the polarity of scCO_2,_ possibility to extract thermolabile compounds at low temperature	High equipment cost, elevated pressure required, risk of volatile compounds losses [54,55]
MAE	High extraction yields, small equipment size, easy industrial escalation, low solvent consumption, possibility to develop a solvent-free process, low power consumption, good reproducibility	High equipment cost, non-selective extraction separation, and purification steps required, very poor efficiency for volatile compounds, lack of studies on modeling of the heating process to improve its uniformity [56,57]
UAE	Significant savings in maintenance, low equipment cost, low operating temperature, efficient extraction of thermolabile compounds	Separation and purification steps required, lack of uniformity in the distribution of ultrasound energy, potential change in the constitutive molecules, large amount of solvent, difficulty in scaling [58]
PEF	Non-destructive, high selectivity, no thermal effect, no need for energy-intensive drying pretreatment, energetically efficient, continuous operability, easy to scale up	Dependence on medium composition (conductivity), high cost of the equipment [59]
HPH	High extraction yields, high scalability, ability to overcome high cell wall rigidity, effective in aqueous environments (eliminating the need for energy-intensive drying), one of the most used mechanical methods for large-scale cell disruption	Non-selective extraction, cell debris can bring downstream complications and costs, temperature increase undesirable for heat-sensitive extracts, cooling needed, high energy consumption [60]
HVED	High extraction yields, efficient extraction of thermolabile compounds, low solvent consumption, low energy consumption, possibility to extract thermolabile compounds	Batch mode operation, hard to be scaled-up, free radicals would be produced leading to oxidative cell damage, but may also oxidize the target compounds, requires precise control of input energy, less selective than PEF [61,62]

**Table 4:** Extraction of bioactive compounds from agri-food by-products using green solvents and/or assisted by non-conventional technologies.

Raw Materials	Target Compounds	Emerging Technology	Green or Sustainable Extraction Approach	Main Findings	Reference
Ionic liquids					
Olive mill wastewater	Tyrosol	/	[P4441] [Tf2N] and 20% wt sodium chloride T = 70 °C, time = 2 h, L/S = 5 mL/g	Extraction efficiencies higher than 94%, comparable to those of conventional organic solvents	[41]
Rice bran	γ-oryzanol	MAE	0.7 M [Bmim]PF6 solution power = 30%, extraction time = 10 min, L/S = 15 mL/g	IL-MAE method more efficient in extracting 0.27 mg/g of γ-oryzanol than the conventional extraction	[83]
			0.7 M [Bmim]BF4 solution power = 30%, extraction time = 10 min, L/S = 15 mL/g	IL-MAE is efficient in extracting γ-oryzanol from rice bran (0.41 mg/g)	[37]
Pomelo peels	Naringin	MAE	10 mmol/L [HO_3_S(CH_2_)4 mim] HSO_4_, power = 331 W, time = 15 min, L/S = 26 mL/g	Enhanced extraction yields of 8.38 ± 0.20 mg naringin/g. Reduction of extraction time from 180 min to 15 min	[84]
Melinjo (Gnetum gnemon L.) seeds	Resveratrol	MAE	2.5 mol/L [Bmim] Br; power = 10%; time = 10 min	The antioxidant activity of IL-MAE melinjo seed extract was 82.82% of DPPH inhibition compared to the one of conventional extraction, which inhibits only 5.96%	[85]
Shrimp waste	Astaxanthin	UAE	[P4448]Br/(TX-100 + n-butanol)/water Ultrasonic power = 50 W, time = 60 min	ILs enhanced the extraction of astaxanthin due to the stronger electrostatic interactions and hydrogen-bonding compared with organic solvents (extraction yield: 99%)	[50]
Orange peels	Carotenoids	UAE	1-butyl-3-methylimidazolium chloride ([BMIM][Cl]), power = 200 W, f = 20 kHz, 80% amplitude, time = 5 min, L/S = 3 mL/g	Total carotenoid content of 32.08 ± 2.05 μg/g using IL, and 7.88 ± 0.59 μg/g using acetone. IL and carotenoid recovery yields using XAD-7HP resin were 59.5–63.8% and 52.2–58.7%	[86]
Deep Eutectic Solvents					
Fig leaves	Caffeoylmalic acid, psoralic acid-glucoside, rutin, psoralen and bergapten	MAE, UAE	Glycerol, xylitol, and D-Fructose (3:3:3 molar ratio) power = 250 W (MAE) and 700 W (US), time = 10 min (MW) and 60 min (US), T = 40–80 °C	Extraction yields: 6.482 mg/g, 16.34 mg/g, 5.207 mg/g, 15.22 mg/g and 2.475 mg/g, respectively, under optimal extraction conditions (64.46 °C, L/S 17.53 min and 24.43 min using UAE)	[87]
Grapefruit peels	Naringin	HVED	Lactic acid:glucose (5:1) HVED as pre-treatment technology (energy 7.27–218 kJ/kg) Solid-liquid extraction T = 50 °C, time = 60 min, L/S = 10 mL/g,	Energy reduction of the HVED pre-treatment by 6 times	[88]
Grape pomace	Anthocyanins	Simultaneous UAE and MAE (UMAE)	ChCl:citric acid with 30% water MAE power = 300 W and UAE power = 50 W, time = 10 min, L/S = 33.33 mL/g	The extraction yield of anthocyanins under optimal conditions is 1.77 mg/g _DW_	[89]
	Polyphenols	UAE	Sodium acetate:lactic acid molar ratio of 5:1, T = 80 °C, time = 90 min, L/S = 30 mL/g	Total polyphenols yield: 134.54 mg GAE/g _DW_	[90]
Onion, tomato, pear, and olive industrial by-products	Polyphenols	UAE	Lactic acid:glucose (5:1) with 15% water L/S = 75 mL/g	Simple, non-expensive, eco-friendly, and robust system. The application to different matrices demonstrated the versatility of the proposed method	[91]
Onion peels	Polyphenols	MAE	ChCl:urea:water (1:2:4) power = 100 W, time = 15.03 min, L/S = 54.97 mL/g	MAE allowed a recovery of bioactive compounds (80.45 mg GAE/g) 1.5 times higher than conventional extraction with 24-fold reduction in extraction time	[92]
Olive pomace	Polyphenols	HAE, MAE, UAE, HHPAE	ChCl:maltose (1:2); ChCl:glycerol (1:2) Homogenate–(HAE), microwave–(MAE), ultrasound–(UAE) or high hydrostatic pressure–(HHPAE) assisted extractions, T = 60 °C, time = 30 min, 12,000 rpm, L/S = 12.5 mL/g	HAE proved to be the best method with extraction efficiency superior to MAE, UAE, and HHPAE	[93]
Spent coffee ground	Chlorogenic acids and flavonoids	UAE	1,6-hexanediol:ChCl molar ratio 7:1 (HC-6) 67.5% *w*/*w*, T = 60 °C, time = 10 min, L/S = 26 mL/g	Significantly higher extraction efficiency compared to conventional methods using water or aqueous organic solvents	[94]
Buckwheat sprouts	Flavonoids	UAE	80% CCTG (CC-based DES composed of triethylene glycol and 20 vol% water), T = 56 °C, time = 40 min, power = 700 W, f = 40 kHz	DES coupled with UAE is a valuable alternative for the green extraction of flavonoids from buckwheat spouts	[95]
Supercritical fluids					
Grape seeds	Polyphenols	SFE	T = 40 °C, P = 80 bar, flow rate = 6 kg CO_2_/h, co-solvent = 20% (*w*/*w*) ethanol-water	Extraction yield of total polyphenols: 7.1 g GAE/100 g dry matter	[96]
Red grape pomace	Polyphenols, volatile fatty acids, polyhydroxyalkanoates, biogas	SFE	T = 40 °C, P = 80 bar, flow rate = 6 kg CO_2_/h, co-solvent = 57% (*w*/*w*) ethanol-water	Extraction yield of total polyphenols: 2.7 g GAE/100 g dry matter	[97]
Wild thyme by-product	Polyphenols	SFE	SFE1 P = 100 bar, T = 40 °C and SFE2 P = 350 bar, T = 50 °C	Promising natural antioxidants and antimicrobial agents in meat processing (0.075 μL/g ground pork patties)	[98]
Tomatoes peels and seeds	Carotenoids	SFE	T = 80 °C, P = 400 bar, flow rate = 4 g CO_2_/min, time = 2 h	Extraction yield: 410.53 mg lycopene/kg, and 31.38 mg β-carotene/kg from peels, 27.84 mg lycopene/kg, and 5.25 mg β-carotene/kg from seeds, on dry weights	[29]
*Castanea sativa* shells	Ellagic acid, epigallocatechin, catechin, caffeic acid derivative	SFE	T = 60 °C, P = 350 bar, CO_2_, 15% (*v*/*v*) ethanol as co-solvent	Extract as promising nutraceutical ingredient and effective scavenger of NO radical and HOCl	[26]
By-products from filter-tea factory (sage herbal dust)	Diterpene polyphenols	SFE	T = 40 and 60 °C, P = 100–300 bar, flow rate = 0.4 CO_2_ kg/h, time = 5 h	SFE process at 283 bar and 60 °C provided the highest extraction yield of the investigated compounds	[99]
Penaeus monodon waste	Astaxanthin	SFE	15% (*v*/*v*) ethanol as co-solvent, T = 56.88 °C, P = 215.68 bar, time = 120 min, flow rate = 1.89 mL CO_2_/min	Recovery yield of 58.50 ± 2.62 µg/g astaxanthin and 12.20 ± 4.16 µg/g free astaxanthin	[100]
Agave bagasse	Antioxidants and saponins	SFE + UAE	10% (*v*/*v*) ethanol as co-solvent, T = 60 °C, P = 300 bar	Antioxidant capacity from 12.18 ± 1.01 to 20.91 ± 1.66 μmol TE/g when using UAE	[101]
Passion fruit seeds and seed cake	Oil and extract with promising antioxidant and antimicrobial activities	SFE	T = 40–50 °C, P = 150, 250 and 300 bar, time = 2.5–3 h, flow rate = 0.5 kg CO_2_/h	The best yields obtained by SFE at 250 bar/40 °C for the seed (27 ± 1%) and by cold maceration (with EtOH–H2O (1/1, *v*/*v*) for the seed cake (6 ± 1%)	[102]
Spent coffee grounds	Oil fraction, antioxidants	SFE	T = 39.85 °C and 59.85 °C, P = up to 50.0 MPa, flow rate = 1.9 × 10^−3^ kg CO_2_/min, co-solvents: isopropanol, ethanol and ethyl lactate	Co-solvents decreased the extraction time to half of that with pure CO_2_ and increased the antioxidant capacity by up to 12.5 times	[103]
Supramolecular solvents					
Coffee wastewater	Caffeine	/	Amphiphile: 1-hexanol or decanoic acid = 2.9–17.1% *v*/*v*, ethanol = 3.8–46.2% *v*/*v*, time = 20 min	Caffeine yield: 54–65 mg/L of wastewater. Good antioxidant activity (up to 53%)	[104]
Coffee cherry pulp	Phenolic and alkaloid compounds	/	Amphiphile: decanoic or octanoic acid, L/S = 4:1 *v*/*w*, time = 5 min	Extraction yield: 3.6 ± 0.3 mg caffeine g^−1^, 0.9 ± 0.1 mg protocatechuic acid g^−1^	[105]
Spent Coffee grounds	Caffeine, 5-CGA, and total phenolic compounds	/	Amphiphile: 1-Hexanol, decanoic acid, 24% *v*/*v* 1-hexanol, 30% *v*/*v* ethanol and 46% *v*/*v* water, time = 1 min	Extraction yield: 3.32 mg caffeine g^−1^; 4.3 mg chlorogenic acid g^−1^; 60.1 mg 5-GAE g^−1^ (Total phenolic compounds)	[106]
Bio-based solvents					
Ethanol					
Apple dust by-product from filter tea factory	Polyphenols and antioxidants	MAE	Ethanol = 40–80% *v*/*v* time = 15–35 min, power = 400–800 W	Best extraction conditions: 15.2 min, ethanol concentration of 40% and microwave irradiation of 400 W	[107]
Tomato pericarps	Nutrient-rich antioxidant ingredients	MAE	Ethanol = 0–100% *v*/*v*, time = 0–20 min, T = 60–180 °C, L/S = 22 mL/g, power = 200 W	Extraction yield of 75.5% and ingredients with high levels of sugars, proteins, phenolics, and flavonoids	[108]
Tomato waste	Trans-lycopene, beta-carotene phenolics and flavonoids	MAE	Ethanol = 95% *v*/*v*, L/S = 20 mL/g, power = 180, 300, 450 W, time = 30, 60 and 90 s	300 W for 60 s was the best condition that gave the high quality for bioactive compounds	[109]
Pineapple waste	Polyphenols, antioxidants	UAE	Ethanol = 0, 20 and 40% *v*/*v*, L/S = 10 mL/g, US mode = 0.5, time = 10, 20, 30 min, power = 200 W	UAE and ethanol as a solvent effective method for the extraction of bioactive compound	[110]
Artichoke wastes	Phenolic compounds	UAE	Ethanol = 50% *v*/*v*, L/S = 10 mL/g, time = 10 min, power = 240 W	UAE favoured the extraction of phenolic compounds, but power > 240 W had no influence on process efficiency	[30]
Peach waste	Total phenolic content, total flavonoid, anthocyanins	UAE, MAE	Ethanol = 70% *v*/*v*, MAE power = 540 W, UAE power = 23%, MAE time = 50 s, UAE time = 120 s	Comparable extraction efficiency. However, vitamin C was successfully extracted only by MAE, due to oxidative degradation during UAE	[111]
Peach waste	Total phenolic content, total flavonoid, anthocyanins	PEF	Ethanol = 70% *v*/*v*, W = 0.0014 kJ/kg, treatment time = 16 μs	PEF led to a reduction of extraction times (16 μs), compared to thermal extraction (40 min), reaching the same yields	[16]
Pomelo peels	Naringin	PEF	E = 4 kV/cm, pulses = 30, L/S = 90 mL/g, solvent = ethanol 40% *v*/*v*, T = 40 °C	PEF improved the extraction yields of naringin by 20% compared with the untreated sample	[21]
Lettuce waste	Polyphenols	HPH, UAE	Ethanol = 50–75% *v*/*v*, HPH: P = 50 MPa, US: P = 400 W, f = 24 kHz, time = 120 s, L/S = 50 mL/g,	HPH led to a reduction in phenolic yields compared to UAE, possibly due to the 40% activation of polyphenol oxidase	[31]
Potato peels	Phenolic acids	HPH	L/S = 25 mL/g in ethanol and NaOH (0–0.4 mol/L), T = 40 °C, P = 158.58 MPa, n 2 passes	The combination of NaOH and HPH improved the extraction yield of total phenolic acid. The highest contribution is associated with HPH	[112]
Fresh rosemary and thyme by-products	Phenolics	PEF pre-treatment, then, UAE	PEF: *n* = 167, pulse width = 30 µ, 0.1% aqueous NaCl, L/S = 1.4 *v*/*w* for rosemary, and 1.5 *v*/*w* for thyme, E = 1.1 ± 0.2 kV cm^−1^, W = 0.36 and 0.46 kJ kg^−1^ for rosemary and thyme US: T = 40 °C, P = 200 W, Ethanol = 55.19% *v*/*v*, L/S = 20 mL/g, time = 12.48 min	PEF pre-treatment enhanced (*p* < 0.05) the recovery of phenolics and antioxidant activity compared to US individually	[51]
Jabuticaba peels	Anthocyanins, pectin	UAE	UAE intensity = 3.7 W/cm^2^, Ethanol = 50% *v*/*v*, L/S = 25 mL/g	The synergy between UAE and the solvent strongly influenced the extraction efficiency of anthocyanins	[113]
Citrus peels	Polyphenols (TPC), flavonoids (TFC)	UAE	70.89% amplitude, L/S = 40 mL/g, time = 35 min	TPC and TFC yield of 1590 ± 0.92 mg GAE/100 g and 104.99 ± 0.35 mg QE/100 g, respectively	[114]
Mushroom stalks	Ergosterol and antioxidant components	UAE	Ethanol = 70 and 96% *v*/*v*, power density = 182 ± 7 W/L, 321 ± 14 W/L, L/S = 5 mL/g	Extraction yield increases up to 2 times in ergosterol, 46% in phenolic compounds, and 25% in antioxidant activity	[36]
Spent coffee grounds	Chlorogenic acid (CGA), protocatechuic acid (PCA)	UAE	Power = 244 W, T = 40 °C, time = 40 min, L/S = 17 mL/g	Extraction yield: 1.34 ± 0.37 mg/g of CGA and 0.51 ± 0.03 mg/g of PCA	[115]
Shrimp shells	Astaxanthin	UAE	L/S = 7 mL/g, time = 20 min, T = 50 °C, f = 40 kHz	Extraction yield is 43.7 g/g. The purity of the obtained astaxanthin was 85.1% using silica gel column chromatography	[116]
Spent coffee grounds	Polyphenols	HVED	Ethanol = 24% *v*/*v*; peak voltage = 11 kV; flow rate = 12 L/h; L/S = 15 mL/g; time = 20 min	Extraction yields are higher by 20.03% than solvent extraction. Reduced extraction time (by 87%) and energy consumption (by 65%)	[27]
Glycerol					
Red grape pomace	Polyphenols, flavonoids	/	T = 23 °C, time = 180 min, S/L = 50 mL/g	Aqueous glycerol (20%, *w*/*v*) is suitable for retrieving polyphenols, flavonoids, and pigments from grape pomace	[117]
		UAE	Power = 140 W, f = 37 kHz, time = 60 min, T = 45 °C, Glycerol = 90% (*w*/*v*), L/S = 90 mL/g	Aqueous glycerol in combination with UAE can efficiently extract polyphenols and pigments	[118]
Potato peels	Polyphenolic antioxidants	UAE	Power = 140 W, f = 37 kHz, time = 90 min, Water/glycerol: glycerol = 83% (*w*/*v*), L/S = 81 mL/g, T = 80 °C Water/ethanol: ethanol = 59% (*v*/*v*), L/S = 84 mL/g, T = 77 °C	Extraction yield in total polyphenols: 8.71 and 9.11 mg caffeic acid/g dry weight, for water/glycerol and water/ethanol mixtures, respectively	[119]
Onion wastes	Polyphenols, flavonoids	UAE	Glycerol = 90% (*w*/*v*), T = 50 °C; time = 60 min; L/S = 90 mL/g	Aqueous glycerol UAE efficiently extracted polyphenols from onion wastes (yield: 90.07 mg GAE/g)	[120]
Spent filter coffee	Polyphenols	UAE	Glycerol 3.6% (*w*/*v*), T = 45 °C; time = 175 min; L/S = 50 mL/g	Aqueous glycerol efficiently provided a higher total polyphenol yield (7.4%) compared to water	[121]
Limonene					
Grape seeds	Fatty acids	/	32% limonene, 35% ethyl acetate, 33% MTBE	The use of limonene allowed obtaining similar yields to longer extraction procedures using organic solvents	[122]
Olive oil					
Tomato peels	Lycopene	/	T = 80 °C, time = 45 min, agitation speeds = 400 rpm, L/S = 0.4% (*v*/*w*)	Extraction yield: 99.3% of the initial lycopene content. Olive oil represents a green solution that prevents lycopene from lipid oxidation	[123]
Water					
Banana peel	Phenolics	MAE	pH = 1, time = 6 min, power = 960 W, L/S = 50 mL/g	Water effectively recovered phenolic compounds (50.55 mg/g dried peel) from banana peel using MAE	[19]
Grape juice waste	Anthocyanins	MAE	Time = 1–5 min, power = 100–600 W, L/S = 10–50 mL/g	Extraction anthocyanin yield: 1.3215 mg/g of grape waste at the power of 435 W, time of 2.31 min, L/S = 19.22 mL/g	[124]
Mango peels	Polyphenols, proteins, carbohydrates	PEF, HVED	Electric field strength (PEF) = 13.3 kV/cm, (HVED) = 40 kV/cm, *n* = 2000, W = 1000 kJ/kg distance between pulses = 2 s, T = 20 °C, L/S = 10 (*w/w*)	HVED is more effective than PEF, however, PEF is more selective	[125]
Fermented grape pomace	Total phenolic compounds, anthocyanins	UAE, PEF, HVED	US: power = 400 W f = 24 kHz PEF: E = 13.3 kV/cm, W = 0–564 kJ/kg, HVED: W = 0–218 kJ/kg, L/S = 10 mL/g	HVED led to the highest phenolic compound’s recovery with lower energy requirement than PEF and US	[62]
Tomato peels	Polyphenols, proteins	HPH	P = 100 MPa, *n* =10 passes, L/S = 10 mL/g	Increase in proteins (+70.5%), polyphenols (+32.2%), antioxidant activity (+23.3%)	[28]
Potato peels	Phenolic compounds	PEF	Pre-treatment: E = 1 kV/cm, W = 5 kJ/kg, treatment time = 6 ms, L/S = 1 mL water/g S/L extraction: Ethanol = 52%, time = 230 min, T = 50 °C	PEF reduced time, temperature, and solvent, improved the extraction yield (10%) and antioxidant activity (9%) than the untreated sample	[34]
Custard apple leaves	Phenolic compounds	PEF	Pre-treatment: E = 2, 4 or 6 kV/cm, W = 45, 94 or 142 kJ/kg, treatment time = 2.5–5 min, L/S = 2.5 mL/g S/L extraction: Ethanol = 70, L/S = 15:1 (*v*/*w*)	PEF improved the extraction yields (+5.2%) and the antioxidant activity than the untreated sample	[126]
Olive pomace	Phenolic compounds	UAE	Power = 250 W, time = 75 min, T = 30 °C, L/S = 50 mL/g	UAE increased the extraction yield of phenolic compounds of 30% compared to the the control	[127]
Sesame cake	Polyphenols, proteins	PEF, HVED	Pre-treatment: E = 13.3 kV/cm, W = 83 kJ/kg, treatment time = 1–7 ms, holding time = 4–28 min, T = 20–60 °C, L/S = 10 mL/g S/L extraction: Ethanol = 10%, L/S = 20 mL/g, time = 1 h	PEF and HVED accelerated the diffusion kinetics, making the impact of temperature smaller	[44]
Pomegranate peel	Phenolic compounds	HVED	T = 25 °C; peak voltage = 9 kV; flow rate = 12 mL/min; L/S = 35 mL/g; electrodes distance = 3.1 mm; time = 30 min	Extraction yield: 196.7 ± 6.4 mg/g. HVED is more efficient in extracting phenolic compounds than the warm water maceration	[128]

**Table 5:** Computational models, predicted physical properties, and their associated advantages and limitations.

Selectivity Criteria	Predicted Properties	Advantages	Limitations	References
Kauri-butanol index	Relative solvency power of a solvent, based on the maximum amount of solvent added to a solution of Kauri gum in n-butanol without causing cloudiness	Simple model	Provides a scaleless index. Not suitable for oils and fats. Sometimes inconsistent with theoretical results. Conducting the test under conditions other than 25 °C, 1 atm yields different results	[177]
Kamlet-Taft scale	Hydrogen bond donation ability (α), hydrogen bond acceptor ability (β), dipolarity-polarizability (π*)	Simple scale-based model. Widely used multiparameter scale	Sometimes inconsistent results	[177,178]
Hildebrand solubility parameters	Interaction degree between chemicals, relative solvency behavior	Simple predictive theory Good indication of solubility, especially for nonpolar or slightly polar systems without hydrogen bonding	Not suitable for polar systems	[179]
HSPs	Total cohesive energy density as the result of the combination of three intermolecular interactions	Powerful indicator of predicted solubility	Physicochemical properties of some “green” solvents are insufficiently investigated. More complicated three-dimensional solubility parameters	[180]
COSMO-RS	Molecular polarity distribution accordingly integrated to calculate the chemical potential of the surface (σ-potential)	Very accurate method, very robust and valuable tool. Applied in a wide range of industrial applications	The quantum chemistry calculation step requires expertise as well as a significant computational time	[181]
